# Use of intra-medullary stacked nailing in the reduction of proximal plastic deformity in a pediatric Monteggia fracture: a case report

**DOI:** 10.1186/1752-1947-5-153

**Published:** 2011-04-16

**Authors:** Jason Lim, James S Huntley

**Affiliations:** 1 University of Glasgow, University Avenue, Glasgow G12 8QQ, UK; 2 Orthopaedic Department, Royal Hospital for Sick Children, Dalnair Street, Yorkhill, Glasgow G3 8SJ, UK

## Abstract

**Introduction:**

In a Monteggia fracture dislocation, it is important to reduce the ulnar fracture completely. Extensive plastic deformation of the proximal ulna may make reduction by closed manipulation impossible.

**Case presentation:**

We report the case of a four-year-old Caucasian boy in whom the plastic deformation of the proximal ulna was reduced, and this reduction was maintained, using intra-medullary stacked nailing.

**Conclusion:**

The technique of stacked nailing is a useful addition to the armamentarium in the management of the potentially awkward Monteggia fracture.

## Introduction

A Monteggia fracture is a fracture of the ulna associated with a radio-capitellar dislocation [1,2]. Pediatric Monteggia injuries, in contrast to those of adults, are usually managed effectively by closed reduction [1]. In a recent one-year series of forearm fractures in Glasgow, Monteggia fracture dislocations accounted for only a minority of injuries (4 *ex *317) [3]. Though uncommon, it is vital to recognize the radio-capitellar dissociation early. The ulnar fracture is usually apparent on clinical and radiological assessment, but up to 50% of radio-capitellar dissociations are missed by senior house officers and 25% are not recognized by senior radiologists [4]. In our center, a review of Monteggia fracture dislocations between 1992 and 2001 showed that about 20% (eight of 39) were initially missed [5]. Adequate treatment is important for achieving good results and to avoid secondary corrective surgery, as missed Monteggia lesions or chronic radial head dislocations may require later reconstruction, which is fraught with potential complications [6].

The current classification of the Monteggia lesion proposed by Bado [2] is widely accepted as standard for adult lesions. The classification scheme of Letts *et al*. [7] for pediatric Monteggia fractures emphasizes the character of the ulnar fracture: A = anterior bend, B = anterior greenstick, C = anterior complete, D = posterior and E = lateral. A stable anatomic reduction of the ulnar fracture usually results in reduction of the radial head [8]. Of the options for the Monteggia fracture dislocation in children, the most common is a manipulative reduction with long-arm cast immobilization in elbow flexion. When the fracture dislocation is unstable or becomes displaced, open reduction and/or internal fixation may be indicated [9-11]. Ring *et al. *[8] also emphasized the importance of the type of ulnar fracture and that plastic deformation of the ulna must be reduced.

De la Garza [12] alluded to the technique of using multiple pins, nesting them within the medullary canal to stabilize the ulna. Ulnar intra-medullary wires can also be used to treat complete transverse and short oblique fractures to prevent angular deformity. These procedures can be done either via an antegrade approach by passing the intra-medullary nail through the olecranon or by using a retrograde approach through the distal ulnar metaphysis. However, if the ulnar fracture is comminuted or has a long, oblique pattern, plate and screw fixation may be required. There may also be a need to remove interposed soft tissue or bony fragments to allow for radial head reduction [10].

Thus it is important to reduce the ulnar fracture, but in patients with extensive proximal plastic deformity, this may prove impossible by manipulation alone. Here we present a case involving the use of a technique that allows for closed reduction and stabilization.

## Case presentation

**Figure 1:**
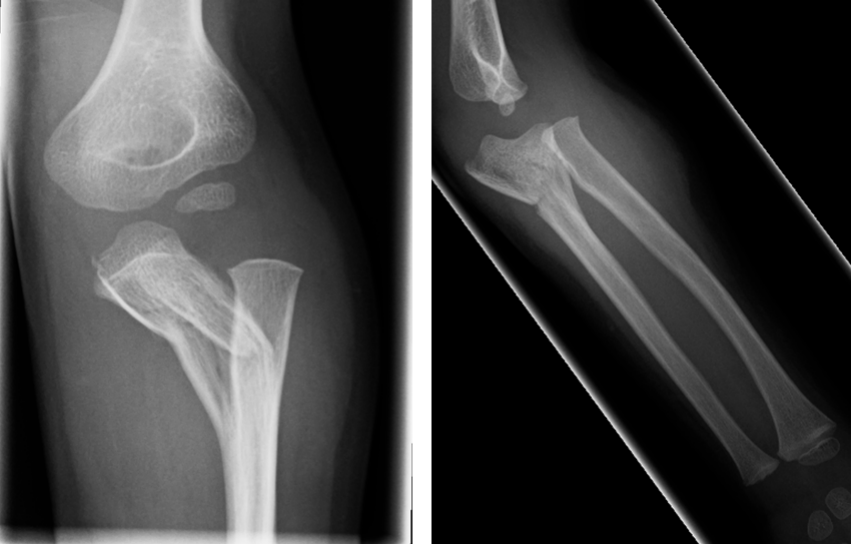
**Injury films showing extensive plastic bowing of the proximal ulna and a radio-capitellar dislocation**.

A four-year-old Caucasian boy with no medical history presented to our emergency department with a right forearm fracture after falling out of a tree. His neurovasculature was intact. Radiography showed a fracture of the proximal ulnar metaphysis with marked varus angulation and dislocation of the radial head both anteriorly and laterally (Figure 1), a combined Bado [2] types I and III Monteggia fracture dislocation. There was no associated distal fracture.

**Figure 2:**
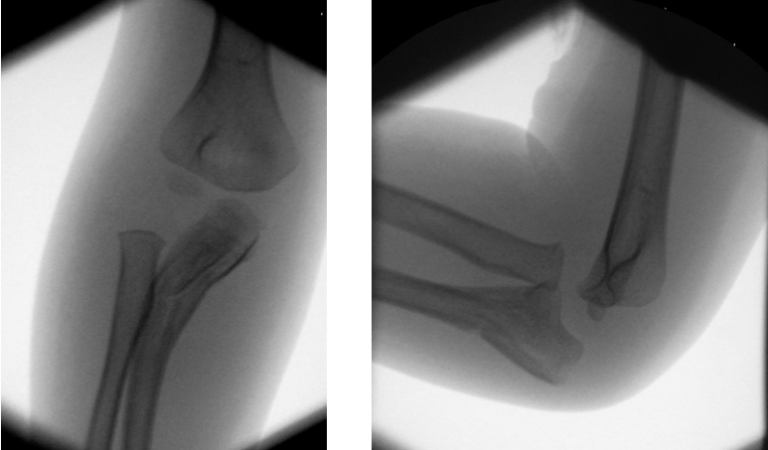
**Image intensifier anteroposterior and lateral views after attempted manipulation of the right elbow**. Although the position is marginally improved, there is still extensive plastic deformation of the ulna as well as radio-capitellar dislocation.

**Figure 3:**
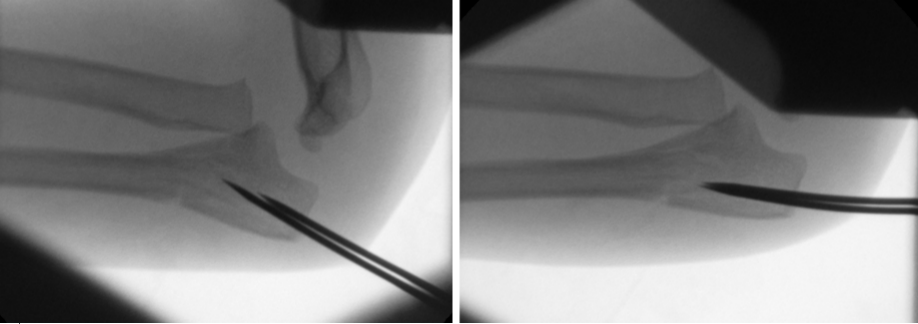
**Image intensifier lateral views showing reduction maneuver using K-wires**. Serial views show the use of two proximal K-wires as a joystick to reduce the proximal ulnar deformity.

**Figure 4:**
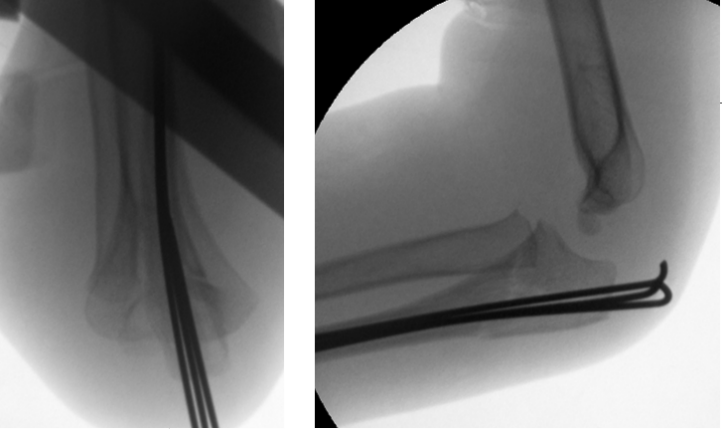
**Image intensifier anteroposterior and lateral views of stacked nailing of the right elbow**. A third K-wire provided an intra-medullary jam fit, which both reduces and stabilizes the Monteggia fracture dislocation.

A closed reduction was attempted on the following day in the surgical theater, but the proximal ulnar fracture was irreducible by manipulation. Therefore, a percutaneous technique using intra-medullary diaphyseal wiring was performed (Figure 2). Initially, a 1 cm incision over the olecranon served as an entry point for two antegrade K-wires into the proximal fragment. These were used as a joystick to reduce the plastic bow of the ulna and were then advanced down the medulla (Figure 3). A third K-wire was inserted in a similar fashion, resulting in a tight intra-medullary fit that reduced the ulna and the radio-capitellar joint concomitantly (Figure 4). A plaster of Paris cast was applied to maintain the right forearm in mid-supination and 100° degree flexion.

**Figure 5:**
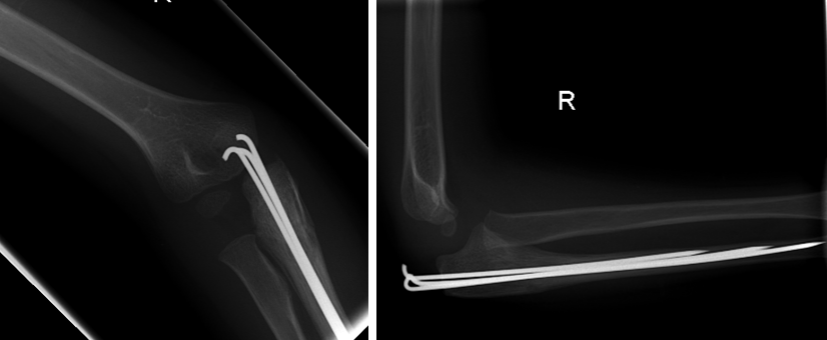
**Anteroposterior and lateral views showing healing of the elbow 6 weeks after the operation**.

Post-operatively, he attended weekly clinic appointments with serial radiographs confirming continued reduction (Figure 5). After the forearm had been immobilized for six weeks, the K-wires were removed with the boy under general anesthesia.

## Conclusions

In our present case report, the problem was one of proximal ulnar bowing with substantial plastic deformity which was irreducible by manipulation alone. Two K-wires inserted longitudinally into the proximal fragment were used as a joystick to partially reduce the ulnar bowing, which was further reduced by passing the K-wires distally. A third K-wire was then added as a jam fit, as is done in the technique of bundle nailing with intra-medullary wires.

The technique described above is similar to that named after Hackethal [13] for his description of stacked nailing as applied to the humerus. Intra-medullary K-wire stabilization is technically easy and minimally invasive. Rabinovich *et al. *[14] suggested that nailing of the skeletally immature ulna can be safely accomplished via antegrade insertion through the olecranon apophysis. Although this approach was successful in our patient with combined Bado types I and III fractures, we have no experience in using this technique in other Bado type fractures (such as the rare type IV fracture, in which there is an associated radial shaft fracture). However, in accordance with the Letts *et al*. classification scheme [7], we suggest that it is largely the character of the ulnar fracture that determines the strategy for reduction and/or fixation; that is, whatever the direction of the radio-capitellar dislocation and whatever associated injuries there are, if there is proximal plastic deformity of the ulna that does not yield to manipulation, then this technique may be useful.

In conclusion, in the context of potentially problematic plastic deformation, we have extended the use of stacked nailing to perform both the reduction and stabilization of a pediatric Monteggia fracture dislocation.

## Abbreviations

AP: anteroposterior; K-: Kirschner

## Consent

Written informed consent was obtained from the patient's parent for publication of this case report and the accompanying images. A copy of the written consent is available for review by the Editor-in Chief of this journal.

## Competing interests

The authors declare that they have no competing interests.

## Authors' contributions

JL wrote the first draft and contributed to the revised manuscript. JH had the idea for the report, revised the manuscript extensively and is the guarantor. Both authors read and approved the final version of the manuscript.
